# The impact of interviewer characteristics on residency candidate scores in Emergency Medicine: a brief report

**DOI:** 10.12688/mep.19735.1

**Published:** 2023-10-04

**Authors:** Ryan F. Coughlin, Jessica Bod, D. Brian Wood, Katja Goldflam, David Della-Giustina, Melissa Joseph, Dylan Devlin, Ambrose H. Wong, Alina Tsyrulnik

**Affiliations:** 1 Department of Emergency Medicine, School of Medicine, Yale University, New Haven, Connecticut, 06510, USA; 2 Department of Emergency Medicine, St. Joseph Medical Center, Dignity Health, Stockton, California, 95204, USA

**Keywords:** Residency selection, Emergency medicine, Interviewer characteristics, Academic rank, Gender, Post-interview discussion, Recruitment, Unconscious bias

## Abstract

**Background:** At the conclusion of residency candidate interview days, faculty interviewers commonly meet as a group to reach conclusions about candidate evaluations based on shared information. These conclusions ultimately translate into rank list position for The Residency Match. The primary objective is to determine if the post-interview discussion influences the final scores assigned by each interviewer, and to investigate whether interviewer characteristics are significantly associated with the likelihood of changing their score. Based on Foucault’s ‘theory of discourse’ and Bourdieu’s ‘social capital theory,’ we hypothesized that interviewer characteristics, and the discourse itself, would contribute to score changes after a post-interview discussion regarding emergency medicine residency candidates.

**Methods:** We conducted a cross-sectional observational study of candidate scores for all candidates to a four-year emergency medicine residency program affiliated with Yale University School of Medicine during a single application cycle. The magnitude and direction of score changes, if any, after group discussion were plotted and grouped by interviewer academic rank. We created a logistic regression model to determine odds that candidate scores changed from pre- and post-discussion ratings related to specific interviewer factors.

**Results:** A total of 24 interviewers and 211 candidates created 471 unique interviewer-candidate scoring interactions, with 216 (45.8%) changing post-discussion. All interviewers ranked junior to professor were significantly more likely to change their score compared to professors. Interviewers who were women had significantly lower odds of changing their individual scores following group discussion (p=0.020; OR 0.49, 95% CI 0.26-0.89).

**Conclusions:** Interviewers with lower academic rank had higher odds of changing their post-discussion scores of residency candidates compared to professors. Future work is needed to further characterize the influencing factors and could help create more equitable decision processes during the residency candidate ranking process.

## Introduction

Given the binding nature of ‘The Match’ in determining residency candidate-program pairings, all parties are incentivized to ensure optimum compatibility during residency recruitment season. Despite this, a validated scoring system to assess residency candidate interview performances does not exist. Interviewers should therefore consider any factors potentially influencing their scores ^1– 6^.

A literature search was performed using all Ovid MEDLINE(R) database entries from 1946 to November 5, 2020, which was the date the search was performed, and did not identify previous investigations that focused on emergency medicine (EM) candidates, nor the impact of a post-interview group discussion. Search terms included: bias, medical school, decision, debrief, interview. A limited number of studies have investigated interviewer characteristics and their possible impact on residency match scores. A recent study reported that there was no significant effect of interviewer sex, faculty academic rank or title on internal medicine candidate scoring ^7^. Another found internal medicine residents involved in interviewing consistently gave candidates more favorable scores than faculty interviewers but including resident interviewers did not lead to a significant impact on initial or final rank list position of candidates ^8^.

The primary objective is to determine if the post-interview discussion influences the final scores assigned by each interviewer, and to investigate whether interviewer characteristics are significantly associated with the likelihood of changing their score. Our hypothesis was based on Foucault’s ‘theory of discourse’ and Bourdieu’s ‘social capital theory’ ^9^. According to the theory of discourse, what a society [in this case, the interviewer group] holds true changes based on the exchange of ideas of those belonging to the society. Social capital theory describes the concept that one’s social position [in this case, interviewer characteristics including academic rank] is a form of resource or commodity that can be used in times of discourse or conflict [in this case, post-interview candidate ranking] ^10^. Therefore, we hypothesized that interviewer characteristics, and the discourse itself, would contribute to score changes.

## Methods

### Ethical considerations

The Yale University School of Medicine Institutional Review Board deemed this deidentified study “Not human research” and exempt from consent requirements (Protocol ID #2000025029, Determined 2/21/2019). Specifically, no consent for publication was required as data has been anonymized, and the data alterations have not distorted scientific meaning.

### Participants

The study was conducted at a four-year Accreditation Council for Graduate Medical Education (ACGME)-accredited EM residency program affiliated with Yale University School of Medicine, which is a quaternary referral center in the United States. Subjects were all faculty and resident interviewers during the complete 2018–19 application cycle (October 10, 2018–January 23, 2019). Residency applicant interviewers included eight senior residents, four chief residents, three clinical instructors, six assistant professors, one associate professor, and two professors of EM. Each candidate had a maximum of one resident interviewer. All interviewers, interviewed applicants, and their interactions were both eligible and included. All 24 interviewers and all 211 candidates were both eligible and included for a total of 471 unique Interviewer-Candidate pairings of scores.

### Data collection

We conducted a cross-sectional, consecutive observational study to determine any change in score that resulted from the discussion session at the end of interview days. The interview structure at the study site included four one-on-one interviews. After each interview, but prior to group discussion, interviewers independently numerically scored each candidate on a scale from 0 to 10. The day concluded with a closed discussion session attended by all interviewers. During this discussion, each candidate’s application and interview performance were reviewed by the entire group, allowing an opportunity for shared perspectives and optional revisions to initial candidate scores. These scores were ultimately used to create the first iteration of the rank list, reviewed again at the final end-of-season discussion. Two scores were obtained for each candidate from each individual interviewer: first immediately following the one-on-one interview, and second following review of the candidate in the closed group discussion. A validated scoring system to assess residency candidate interview performances does not exist, but this method differs from the historical scoring of a single score after the closed group session, that is anecdotally edited with some frequency after discussion but before submission. Closed group discussions last approximately 10 minutes per candidate.

Data were collected by one physician author (man) and the dataset was de-identified and coded by the residency program coordinator (non-physician woman), who did not participate in interviews nor the composition of any portion of this manuscript and entered into a Microsoft Excel (RRID:SCR_016137) worksheet. Data from interviews conducted by the physician author who collected the data were not included in the analysis. Interviewers included peers, educators, advisees, and supervisors of the physician data collector. All participants were aware of data collection and its purpose. Gender identification was by self-report. No interviews occurred more than once. No prompts were provided by authors, no audiovisual recording was used in data collection. No notes other than scores were taken, and corrections of scores after final submission were not allowed. Statistical calculation revealed that our dataset was appropriately powered to draw mathematical conclusions.

### Statistical analysis

We determined the odds of candidate scores changing before and after discussion as related to specific interviewer factors using logistic regression modeling. The following variables were included in the model: interviewer academic rank, interviewer sex, score prior to the discussion, and candidate final rank group. A total of 454 candidate interviews were included in the analysis. A p-value of <.05 was chosen as statistically significant. We used IBM SPSS Statistics (RRID:SCR_016479) software (v. 22.0, IBM Corp) to perform statistical analyses. No funding was obtained during this undertaking.

## Results

In total, 216 (45.8%) scores changed from pre- to post-discussion. Logistic regression results are summarized in Table 1 ^11^.

**Table 1.** OR of interviewers changing candidate scores following group discussion. (Abbreviations: n = number; OR = odds ratio; CI = confidence interval).

Variable	n	Adjusted OR (95% CI)	p-value
Interviewer Gender			
Men	12	Reference value	──
Women	12	0.49 (0.26, 0.89)	0.020
Interviewer Rank			
Professor	2	Reference value	──
Associate Professor	1	9.56 (2.60, 25.40)	<0.001
Assistant Professor	6	12.78 (5.47, 29.87)	<0.001
Instructor	3	4.31 (2.02, 9.18)	<0.001
Chief Resident	4	9.55 (3.92, 23.24)	<0.001
Resident	8	4.94 (2.16, 11.33)	<0.001
Candidate Rank List Group			
Bottom Third		Reference value	──
Middle Third		0.34 (0.20, 0.59)	<0.001
Top Third		0.26 (0.14, 0.48)	<0.001
Score Prior to Discussion		1.15 (1.02, 1.31)	0.029

**Figure 1.**
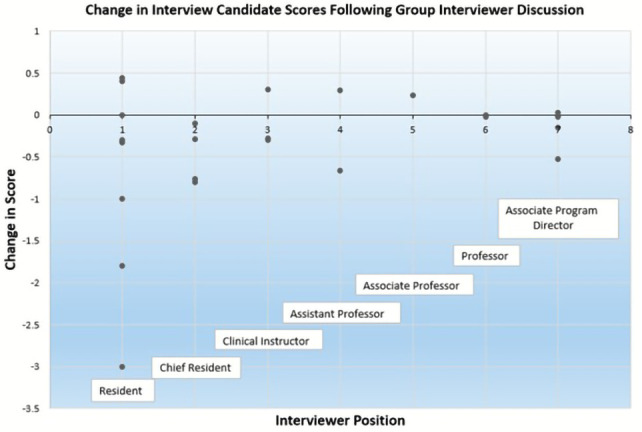
Change in interview candidate scores following group interviewer discussion.

All interviewers ranked below professor were significantly more likely to change their score as compared to professors. Candidates in the top two thirds of the ultimate rank list were less likely to have their score changed post-discussion as compared to the bottom third (top third: OR 0.26 (95% CI 0.14, 0.48); middle third: OR 0.34 (95% CI 0.20, 0.59)). Interviewers who were women had significantly lower odds of changing their individual scores following group discussion (OR 0.49 (95% CI 0.26, 0.89)) as compared to men. For graphical representation of the degree and direction of candidate score change after discussion, please see Figure 1.

## Discussion

Post-interview discussion resulted in an increased likelihood of score changes by all interviewers, except for full professors. One of the full professors in the study was the residency program director (PD), who has a supervisory role over every interviewer within the department, except for the other full professor. Evaluating these results on the basis of Foucault’s ‘theory of discourse,’ the findings could be explained by the idea that power structures of the post-interview discussion had an influence in the outcomes: the junior faculty and resident interviewers could have a conscious or unconscious desire to ‘agree’ with their supervisor ^9^. Alternatively, they may simply have adjusted their score based on reconsideration of the candidate considering other interviewer perspectives. Another background consideration rooted in the ‘social capital theory’ is that residents and junior faculty are closer to candidates in career progression, and so may theoretically have an easier time relating to applicants, whereas senior ranking faculty will have more life, clinical, and interviewing experience, though also be less likely to be affected by the impressions of more senior faculty ( *i.e.*, the one full professor did not change their rank) ^10^. Both interviewer groups use their social context to rate the candidate, thus scores change after the discussion occurs.

In future investigations it would be interesting to include candidate demographics and non-academic-rank groupings of faculty such as years of practice. Further work could also examine whether score changes may be more likely to affect final rank list positions for particularly strong or particularly weak candidates more than those in the middle.

Given the stakes for all parties involved in the residency matching process, it is particularly important that interviewers consider all variables, including the influence of group discussion observed here, that may affect a candidate’s position on the rank list. This is especially true of senior faculty who must consider their potential influence on junior interviewers, while junior interviewers should recognize their possible vulnerability to this influence.

A larger conversation by national organizations may be warranted regarding the positive and negative aspects of subjective numerical candidate ranking systems and complexities of biases, which are among the challenges encountered in some of the current selection processes.

In addition to a small sample size, there were very few professors and associate professors interviewing during the investigation time window. A replication of this study could also consider using years of interviewing experience as a seniority variable instead of academic ranking.

Interviewer reasoning for score adjustment was not evaluated in this study. Further investigation of score change rationale may clarify the influences on their decisions, though could also be influenced by reporting bias. It would be noteworthy to study any difference in reasoning between gender groups, as there was a significant difference in score change in our study.

The role of candidate socioeconomic status, race, ethnicity, and gender identity, which have been investigated in medical school interviews as well as several other industries, were not addressed in this study but could be an area for future investigation regarding candidate characteristics and any association with interview scores ^7, 8, 12^.

## Conclusions

Interviewers with lower academic rank had higher odds of changing their post-discussion scores of residency candidates compared to those at the professor level. Future work is needed to further characterize the influencing factors and could help create more equitable decision processes during the residency candidate ranking process.

## Data Availability

Zenodo: Deidentified Data Set For The Impact of Interviewer Characteristics on Residency Candidate Scores. https://doi.org/10.5281/zenodo.8172929 ^11^. This project contains the following underlying data: Deidentified Data.xlsx (restricted access) This data may make individuals identifiable even without a data dictionary due to recorded characteristics of involved parties and the dataset pertains to a confidential interview process and surrounding the confidential residency match process, which should not be shared. Though references to individuals are indirect, publishing the data could make individuals identifiable and is also a violation of the signed agreement referenced below. See section ‘6.4 Confidentiality’ from the NRMP website ( https://www.nrmp.org/wp-content/uploads/2022/09/2023-MPA-Main-Match-Program-FINAL-3.pdf) regarding confidentiality from the Match Participation Agreement Program: 2023 Main Residency Match and Supplemental Offer and Acceptance Program (SOAP). Data access may be obtained by submitting an electronic request to the corresponding author of The Impact of Interviewer Characteristics on Residency Candidate Scores in Emergency Medicine A Brief Report. All requests will be reviewed by the authors before being allowed.
