# Supplementary material for: The impact of interviewer characteristics on residency candidate scores in Emergency Medicine: a brief report
Source: MedEdPublish (2016). 2024 Feb 13;13:205. Originally published 2023 Oct 4. [Version 2] doi: 10.12688/mep.19735.2 (PMC10933563; doi:10.12688/mep.19735.2)
Supplement: Peer review history

# Reviewer report: Reviewer response for version 2

**DOI:** 10.21956/mep.21645.r36015

**Published:** 2024-03-12

**Authors:** Christie Lech

**Affiliations:** 1 Weill Cornell Medicine, New York, NY, USA

No new comments to add

Have any limitations of the research been acknowledged?

Partly

Is the study design appropriate and does the work have academic merit?

Partly

Is the work clearly and accurately presented and does it cite the current literature?

Yes

If applicable, is the statistical analysis and its interpretation appropriate?

Yes

Are all the source data underlying the results available to ensure full reproducibility?

Yes

Are the conclusions drawn adequately supported by the results?

Yes

Are sufficient details of methods and analysis provided to allow replication by others?

Yes

Reviewer Expertise:

Medical education

I confirm that I have read this submission and believe that I have an appropriate level of expertise to confirm that it is of an acceptable scientific standard.

# Reviewer report: Reviewer response for version 2

**DOI:** 10.21956/mep.21645.r36016

**Published:** 2024-03-06

**Authors:** Milad Memari

**Affiliations:** 1 Department of Medicine, University of Virginia, Charlottesville, Virginia, USA

This is an important study attempting to understand the potential social capital that factors such as academic rank and gender may have on changes made to interviewee scoring during a post-interview group review. The study question and background is appropriate, supported by theory and well-articulated. In particular, the authors' point in the discussion that the interviewing and ranking process is complex and bears further conversation is an important one and I applaud the authors for attempting to investigate this further with a rigorous approach.

 The authors have evidence supporting the assertion that scores by junior faculty are more likely to change. The question remaining for me is whether the senior evaluators (including the PD who is one of the professors) have post-hoc opportunity to influence the rank list, thereby making them less likely to change the actual score in the first iteration of the rank list upon which the analysis is based. The authors mention a final "end-of-season discussion" which may allow further modification. As such, if the discussion session is seen an information-gathering exercise for the PD and other senior faculty who may have a later opportunity to state their views when compared to the more junior interviewees, the measured difference may be less meaningful. Additional limitations in this study are the small number of faculty involved and the single institution nature of the study limiting generalizability. These types of complexities are difficult to account for in a limited setting with a small number of faculty in general.

 I fully agree with authors' suggestion for further studies to evaluate the impact of bias and discussion on rankings for all candidates. This study, despite limitations in generalizability, is helpful as an exploration into this important area of study. Given the fact that these discussions and ranking adjustment processes are likely universal in residency training programs, more studies like this and attempts at understanding the impact of social dynamics on interviewee evaluations is needed. 

**Is the work clearly and accurately presented and does it cite the current literature?**

 Yes

**Is the study design appropriate and does the work have academic merit?**

 Yes

**Are sufficient details of methods and analysis provided to allow replication by others?**

 Partly

**If applicable, is the statistical analysis and its interpretation appropriate?**

 Defer to statistician

**Have any limitations of the research been acknowledged?**

 Yes

**Are all the source data underlying the results available to ensure full reproducibility?**

 Yes

**Are the conclusions drawn adequately supported by the results?**

 Yes

Have any limitations of the research been acknowledged?

Yes

Is the study design appropriate and does the work have academic merit?

Partly

Is the work clearly and accurately presented and does it cite the current literature?

Yes

If applicable, is the statistical analysis and its interpretation appropriate?

No

Are all the source data underlying the results available to ensure full reproducibility?

Partly

Are the conclusions drawn adequately supported by the results?

No

Are sufficient details of methods and analysis provided to allow replication by others?

Partly

Reviewer Expertise:

Medical Education, Learner Development, Growth Mindset, Coaching

I confirm that I have read this submission and believe that I have an appropriate level of expertise to confirm that it is of an acceptable scientific standard, however I have significant reservations, as outlined above.

# Reviewer report: Reviewer response for version 1

**DOI:** 10.21956/mep.21143.r35732

**Published:** 2024-01-30

**Authors:** Laura R Hopson

**Affiliations:** 1 Department of Emergency Medicine, University of Michigan, Ann Arbor, Michigan, USA

This is an interesting study, and I will admit that I have contemplated (but never executed) a similar approach to understanding the role of group dynamics in the assignment of interview scores. I appreciate the anchoring of the study in theory and the attempt to explain behaviors.

 There are several areas that I believe would benefit from additional attention from the authors in order to present a well-rounded perspective on their data and its analysis.  

 First, these interactions are, almost definitely given their dates, in person interactions rather than virtual interactions for both the interview and scoring discussion. This has the potential to be a significant limitation given that their analysis relies on social interactions.

 I agree that a single "validated scoring system does not exist" for residency interviews as noted in the introduction. However, one such tool was published for EM in PMID: Hopson *et al. *(2019 ^1^) (self-citing). There are also strategies that can increase reliability such as behaviorally based interview questions and behaviorally anchored scoring systems. In addition, holistic review allows programs to design interview assessments to map to program priorities. A discussion of these would add richness to the discussion at a minimum. I would also appreciate seeing the scoring tool shared and a brief discussion of its development with the validity evidence behind it to strengthen this work.

 Data collection processes are clearly reviewed and appear appropriate. The statistical analysis generally appears appropriate. However, I am worried there may be significant missing data and would like to see how this data was treated. 4 interviews per candidate with 211 candidates should yield almost 850 data points rather than the 471 included. I would like to see either clarification of this or accounting for an almost 50% loss of data. 

 While it may be beyond the scope of this manuscript, there are some interesting nuances in the data which may merit further investigation - that is the marked variability in response of group members to change which appears to be particularly prominent among the residents and chief residents in Figure 1. In addition, the degree of influence of the discussion is not linearly related to academic rank. Both of these make me wonder if there is another confounder such as years of interview experience which could be involved.

 The authors also have the potential to propose interventions to mitigate the influence of senior members during discussion and I would like to see them add that to this report in the discussion.

Have any limitations of the research been acknowledged?

Partly

Is the study design appropriate and does the work have academic merit?

Partly

Is the work clearly and accurately presented and does it cite the current literature?

Partly

If applicable, is the statistical analysis and its interpretation appropriate?

Yes

Are all the source data underlying the results available to ensure full reproducibility?

No source data required

Are the conclusions drawn adequately supported by the results?

Partly

Are sufficient details of methods and analysis provided to allow replication by others?

Yes

Reviewer Expertise:

Medical Education including residency selection processes, the match environment, trainee assessment, and development of expertise.

I confirm that I have read this submission and believe that I have an appropriate level of expertise to confirm that it is of an acceptable scientific standard, however I have significant reservations, as outlined above.

## References

[1] : Comparison of the Standardized Video Interview and Interview Assessments of Professionalism and Interpersonal Communication Skills in Emergency Medicine. *AEM Educ Train* .2019;3(3) : 10.1002/aet2.10346 259-268 10.1002/aet2.10346 31360819 PMC6637001

# Response

**Published:** 2024-02-05

**Authors:** Ryan Coughlin

**Affiliations:** School of Medicine, Dept of Emergency Medicine, Yale University, New Haven, Connecticut, USA

Thank you for taking the time to review our work.  A new version has been submitted.  You have many valid points.  We have added the in-person qualifier in the background and discussion.  We have added the behavioral discussion items and your citation to the Discussion section. We have added an explanation of the missing data points in the Methods section. The data from the program director and the faculty member running the study and collecting data were excluded.  Not including the PD data was suggested by the statistician as it virtually identical to the ultimate final rank list. We agree that years of experience **interviewing** is almost certainly important and attempt to address this in our Discussion section.

# Reviewer report: Reviewer response for version 1

**DOI:** 10.21956/mep.21143.r35735

**Published:** 2024-01-30

**Authors:** Milad Memari

**Affiliations:** 1 Department of Medicine, University of Virginia, Charlottesville, Virginia, USA

This is an important study attempting to understand the potential social capital that factors such as academic rank and gender may have on changes made to interviewee scoring during a post-interview group review. The study question and background is appropriate, supported by theory and well-articulated.

 There is a question as to whether the methods allow for the study question to be adequately addressed. Given the fact that the more senior evaluators (including the PD who is one of the professors) may have more insight into each candidate when compared to the more junior interviewees, there are alternative explanations to the differences in rating changes. There may be other reasons why the more senior faculty do not change ratings. It is also difficult to draw conclusions when comparing small numbers (in this case, an "n" of 2 full professors). 

 Additionally, the statistical description is not fully clear and requires further explanation. The methods state that a logistic regression is reported to have been used without further explanation. The adjusted OR reported are all within the 95% confidence interval for all of the analyses cited and therefore would presumably not reject the null hypothesis in this instance. Unless I am missing something, it is quite unclear how these are statistically significant outcomes. This is altogether not surprising given the fact that the comparison is potentially underpowered. 

 This is an important study and would be interesting if expanded to include more individuals such that a statistically significant outcomes could be determined. As it stands, it is unclear what to take away from this manuscript given the above limitations and whether the authors' conclusions can be accepted.

Have any limitations of the research been acknowledged?

Yes

Is the study design appropriate and does the work have academic merit?

Partly

Is the work clearly and accurately presented and does it cite the current literature?

Yes

If applicable, is the statistical analysis and its interpretation appropriate?

No

Are all the source data underlying the results available to ensure full reproducibility?

Partly

Are the conclusions drawn adequately supported by the results?

No

Are sufficient details of methods and analysis provided to allow replication by others?

Partly

Reviewer Expertise:

Medical Education, Learner Development, Growth Mindset, Coaching

I confirm that I have read this submission and believe that I have an appropriate level of expertise to state that I do not consider it to be of an acceptable scientific standard, for reasons outlined above.

## References

[1] : Statistical inference by confidence intervals: issues of interpretation and utilization. *Phys Ther* .1999;79(2) :186-95 10029058

# Response

**Published:** 2024-02-05

**Authors:** Ryan Coughlin

**Affiliations:** School of Medicine, Dept of Emergency Medicine, Yale University, New Haven, Connecticut, USA

Thank you for taking the time to review our work. A new version has been submitted. We agree that our small sample size and experience of senior evaluators are important limitations.  We attempted to include these items in the third-to-last paragraph of the Discussion section. We have edited the Methods section to better describe our data set. Regarding the results listed in Table 1, there is indeed a statistically significant difference between the listed groups, allowing us to reject the null hypothesis.

# Reviewer report: Reviewer response for version 1

**DOI:** 10.21956/mep.21143.r34993

**Published:** 2023-10-16

**Authors:** Christie Lech

**Affiliations:** 1 Weill Cornell Medicine, New York, NY, USA

It is important to also comment on how the interviewers were trained regarding interviewing of candidates and how they were trained to score the candidates, as this is also an area for potential bias. In addition, years of experience may come into play. Also can touch more on the significance of this work to future outcomes like matching of highly ranked residents etc.

Have any limitations of the research been acknowledged?

Partly

Is the study design appropriate and does the work have academic merit?

Partly

Is the work clearly and accurately presented and does it cite the current literature?

Yes

If applicable, is the statistical analysis and its interpretation appropriate?

Yes

Are all the source data underlying the results available to ensure full reproducibility?

Yes

Are the conclusions drawn adequately supported by the results?

Yes

Are sufficient details of methods and analysis provided to allow replication by others?

Yes

Reviewer Expertise:

Medical education

I confirm that I have read this submission and believe that I have an appropriate level of expertise to confirm that it is of an acceptable scientific standard, however I have significant reservations, as outlined above.

# Response

**Published:** 2024-02-05

**Authors:** Ryan Coughlin

**Affiliations:** School of Medicine, Dept of Emergency Medicine, Yale University, New Haven, Connecticut, USA

It is important to also comment on how the interviewers were trained regarding interviewing of candidates and how they were trained to score the candidates, as this is also an area for potential bias. In addition, years of experience may come into play. Also can touch more on the significance of this work to future outcomes like matching of highly ranked residents etc.  Thank you for taking the time to review our work.  A new version has been submitted. We have added a sentence regarding scoring training in the first paragraph of the Methods Section. We agree that years of experience would be interesting and important and have attempted to include it in the 3rd to last paragraph of the Discussion Section.
